# Psychological symptoms and the MCID of the DASH score in shoulder surgery

**DOI:** 10.1186/s13018-018-0949-0

**Published:** 2018-10-04

**Authors:** Rinco C T Koorevaar, Ydo V Kleinlugtenbelt, Ellie B M Landman, Esther van ‘t Riet, Sjoerd K Bulstra

**Affiliations:** 1 Department of Orthopedics, Deventer Hospital, N. Bolkesteinlaan 75, 7416 SE Deventer, The Netherlands; 2 Teaching Hospital/Research Department, Deventer Hospital, N. Bolkesteinlaan 75, 7416 SE Deventer, The Netherlands; 3 Department of Orthopedics, University Medical Center Groningen, University of Groningen, PO box 30.001, 9700 GB Groningen, The Netherlands

**Keywords:** Psychological symptoms MCID, DASH shoulder surgery

## Abstract

**Background:**

Psychological symptoms are frequently present in patients scheduled for shoulder surgery. The perception of functional disability, activity level and pain in the shoulder is negatively influenced by psychological symptoms, which leads to higher scores of the Disabilities of the Arm, Shoulder and Hand (DASH) questionnaire. The aim of this study was to determine the influence of psychological symptoms on the minimal clinically important difference (MCID) of the DASH score in patients after shoulder surgery.

**Methods:**

In this prospective longitudinal cohort study, 176 patients were included. Group 1 (32 patients) had symptoms of psychological disorders before and after surgery; group 2 had no symptoms of psychological disorders (110 patients). In the remaining patients (34 patients), psychological disorders changed after surgery. Clinical outcome was measured with the change of DASH score and anchor questions for perceived improvement of pain and function after surgery. Symptoms of psychological disorders were identified with the Four-Dimensional Symptom Questionnaire. An anchor-based mean change score technique was used to determine the MCID of the DASH score.

**Results:**

DASH scores before and 12 months after shoulder surgery were significantly higher in patients with symptoms of psychological disorders; change of DASH score was not different between the two groups. The MCID of the DASH score was 13.0 [SD 20.7] in the group with symptoms of psychological disorders and 12.7 [SD 17.6] in the group with no symptoms of psychological disorders. We observed no difference (*p* = 0.559) in the MCID between the group with and the group without symptoms of psychological disorders.

**Conclusion:**

Symptoms of psychological disorders had a negative effect on the DASH score but no influence on the MCID of the DASH score. The DASH score could be used in future studies to assess the influence of psychological factors on the clinical outcome of treatment.

## Background

Psychological symptoms are frequently present in patients scheduled for shoulder surgery [1–3].

The influence and relationship of psychological symptoms with clinical outcome after shoulder surgery has been studied but not clearly defined. Psychological symptoms may have a role in the etiology, perceived disability and pain and the outcome of treatment of shoulder complaints. The Disabilities of the Arm, Shoulder and Hand questionnaire (DASH) is one of the most frequently used PROMs for the shoulder. The perception of functional disability, activity level and pain in the shoulder is negatively influenced by psychological symptoms, which leads to higher DASH scores [3–6]. The minimal clinically important difference (MCID) is defined as the smallest measured change score that patients perceive to be important [7–9]. The MCID of the DASH score has been assessed in the general shoulder population [8–16].

To our knowledge, it is unknown if psychological symptoms influence the magnitude of the MCID of the DASH score. In order to interpret the DASH change score after treatment of shoulder symptoms in patients with and without psychological symptoms, it seems important to assess if the MCID is different in patients with psychological symptoms compared to patients with no psychological symptoms.

The aim of this study was to determine the influence of psychological symptoms on the MCID of the DASH score in patients treated with shoulder surgery. Our hypothesis was that psychological symptoms have a negative impact on the magnitude of the DASH score before and after shoulder surgery and that the MCID will be different in patients with and without psychological symptoms.

## Methods

### Design and study population

This study was a prospective longitudinal cohort study. We included all consecutive patients that were planned for elective shoulder surgery in a one and a half year period (January 2012 until May 2013). Operating procedures were carried out in a general teaching hospital by a single surgeon and his supervised trainees. Patients were considered eligible for the study if they were scheduled for elective shoulder surgery and were at least 16 years of age. Exclusion criteria were diagnostic shoulder arthroscopy and shoulder arthrodesis and unable to complete questionnaires because of language or cognitive disorders. If patients were re-operated or sustained a shoulder fracture within the follow-up period, they were excluded. Participants were informed about the study using a patient information letter and patients had the opportunity to ask questions about the study. Then, informed consent was received orally and formally recorded. Approval for this study was obtained from the Regional Medical Ethical Committee Isala Hospital, Zwolle, the Netherlands, number 14.11151.

**Fig. 1:**
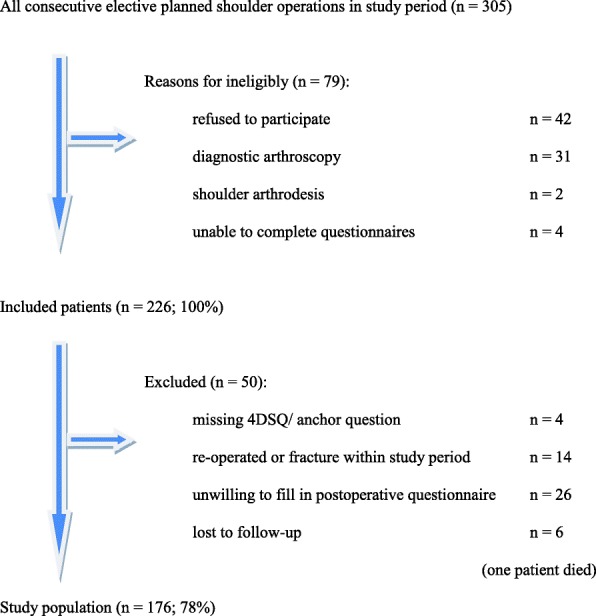
Flow diagram with study enrolment and follow-up

Figure 1 presents a flow diagram with study enrolment and follow-up. In the study period, 205 patients were included. Twenty-three patients (11.6%) were unwilling to fill in the postoperative questionnaire and completed a telephone interview with our research nurse including the anchor questions. Six patients were lost to follow-up; one of these patients died in the study period, which was not related to the shoulder operation. Preoperative DASH score and patients’ perceived improvement of pain and function after shoulder surgery (anchor questions) were not different in the group who completed a telephone interview compared to the group who filled in the postoperative questionnaire.

### Measurements

Prior to elective shoulder surgery, orthopaedic patients were seen 2 to 3 weeks before surgery at an outpatient clinic by an independent physiotherapist from our shoulder unit. Demographic and clinical variables including the DASH and Four-Dimensional Symptom Questionnaire (4DSQ) were obtained. The orthopaedic surgeons and physiotherapists involved in the treatment of the patient were blinded to the results of the psychological questionnaire (4DSQ) to minimize bias. After 1 year, data were obtained using a web-based system. The patients completed an online questionnaire at home containing the DASH, 4DSQ, and the anchor question pain and function. If the patient did not respond to our request to fill in the postoperative questionnaire, a telephone interview by an independent research nurse was conducted with the two anchor questions about pain and function. All data were collected independently by the research unit of our Orthopaedic Department, using standardized case report forms and a study-specific database.

### Outcome measures

#### The Disabilities of the Arm, Shoulder and Hand (DASH) score

The DASH is a 30-item self-report questionnaire designed to measure physical function and symptoms in people with musculoskeletal disorders of the upper limb [17]. The DASH questionnaire has been shown to be reliable, valid and responsive in patients with shoulder disability [18] and has been validated in Dutch for patients with a disorder of the upper limb [19].

### Four-dimensional symptom questionnaire (4DSQ)

The 4DSQ is a psychological questionnaire validated in orthopedic shoulder patients [1]. The 4DSQ is a 50-item self-report questionnaire that identifies four psychological disorders: distress, depression, anxiety and somatisation [20]. The distress scale measures people’s most general, most basic response to stress of any kind, be it work or family demands, psychosocial difficulties or life events. The depression and anxiety scales identify specific symptoms of depressive and anxiety disorders that are severe enough to warrant specific treatment. The somatisation scale measures symptoms associated with somatic stress. Psychological disorders in this study were defined to be present if the patients scored medium or high risk on (one of the items of) the 4DSQ [20, 21]. The 4DSQ was equally able to detect depressive and anxiety disorders as the Hospital Anxiety Depression Scales [21]. A Dutch and an English version of the 4DSQ have been validated [22].

### Anchors

An anchor is a global rating scale in which patients are asked in a single question to indicate how much their function (functional anchor) or pain (pain anchor) has changed since baseline [7, 23]. The response options are completely recovered (7), much improved (6), slightly improved (5), unchanged (4), slightly worse (3), much worse (2) and worse than ever (1). Specific instructions to the patients were to try to remember how painful and how limited their shoulder function was before the surgery and how has the pain or functioning of their shoulder changed compared to the first time they completed this questionnaire.

### Statistical analysis

The DASH change scores were calculated by subtracting each patient’s 12 months after surgery score from the baseline (before surgery) score and were then used to determine the MCID using an anchor-based mean change score technique [24, 25]. There are different measurement techniques to calculate the MCID of the DASH score [26]. We chose the anchor-based mean change method because this method is most frequently used [8–10, 12, 13]. The anchor scores were used to categorize patients into seven subgroups, varying from completely recovered to worse than ever. DASH change scores were calculated in each of the seven subgroups. The MICD was defined as the mean change score in the subcategory of patients who were ‘slightly improved’ according to the anchor scores [7, 24]. The DASH score primarily assesses shoulder function; therefore, we compared these change scores only to the functional anchor. We performed independent samples *t* tests to compare two groups: group 1 included patients with one or more psychological disorders before and 12 months after surgery and group 2 included patients with no psychological disorders before and 12 months after surgery. We decided not to study patients with a change of psychological disorders after surgery because in these patients, only one of the DASH scores before or after surgery was influenced by the psychological disorders, maybe confounding possible associations.

## Results

The total study population consisted of 176 patients; 110 patients did not have any psychological disorders before or after surgery (group 1) and 32 patients had psychological disorders both before and after surgery (group 2). In the remaining patients (34 patients), psychological disorders changed after surgery. These patients were excluded from the analysis. Preoperative psychological disorders disappeared after surgery in 18 patients, and new psychological disorders were observed in 16 patients. Demographic and clinical data of group 1 and group 2 are presented in Table 1.

**Table 1:** Demographic and clinical data of patients in group 1 and group 2

	Group 1	Group 2	*p* value
	*N* = 32	*N* = 110	
Mean age (year; SD)	57.7 (13.7)	50.7 (15.5)	*p* = 0.017
Male gender (no. [%])	10 (31%)	70 (64%)	*p* = 0.002
Duration of symptoms (months; SD)	35.2 (42.7)	34.0 (51.4)	*p* = 0.893
History of surgery (no. [%])	7 (22%)	10 (9%)	*p* = 0.004
Diagnosis:			*p* = 0.042
Subacromial pain syndrome *n* = 17	3 (9%)	9 (8%)	
Rotator cuff rupture *n* = 68	13 (41%)	41 (37%)	
Glenohumeral instability *n* = 39	3 (9%)	30 (27%)	
AC osteoarthritis *n* = 20	2 (6%)	16 (15%)	
Glenohumeral osteoarthritis *n* = 29	10 (31%)	13 (12%)	
Frozen shoulder *n* = 3	1 (3%)	1 (1%)	
DASH preop	55.5 (19.8)	35.3 (21.2)	*p* < 0.001
DASH postop	34.8 (20.5)	12.1 (12.1)	*p* < 0.001
DASH change	−20.7 (21.7)	−23.3 (22.1)	*p* = 0.559

Group 1: preoperative and postoperative with one or more psychological disorders. Group 2: preoperative and postoperative with no psychological disorders

In group 1, significantly more females were present (*p* = 0.002), patients were older (*p* = 0.017), patients more frequently had a history of previous surgery (*p* = 0.004) and more often had glenohumeral osteoarthritis as the primary diagnosis (*p* = 0.042). Glenohumeral instability was less frequently encountered and duration of symptoms were not different between the two groups. DASH scores before and 12 months after shoulder surgery were significantly higher in patients with symptoms of psychological disorders (before surgery: patients with psychological disorders DASH score 55.5 [SD 19.8], patients without psychological disorders DASH score 35.3 [SD 21.2] (*p* < 0.001); 12 months after surgery: patients with psychological disorders DASH score 34.8 [SD 20.5], patients without psychological disorders DASH score 12.1 [SD 12.1] (*p* < 0.001)). Change of DASH score was not different (*p* = 0.559) between the two groups. Previous shoulder surgery and the distribution of shoulder diagnoses could not explain the difference in DASH scores between the two groups. Symptoms of psychological disorders were encountered in all various shoulder diagnoses (Table 2).

**Table 2:** Psychological disorders and change of DASH score in various shoulder diagnoses (*n* = 176)

Diagnosis	Group 1^1^ (*n*; %)	Group 2^2^ (*n*; %)	Others^3^ (*n*; %)	DASH preop	DASH postop	Change of DASH score (mean; SD)
	*n* = 32	*n* = 110	*n* = 34			
Subacromial pain syndrome *n* = 17 (10%)	3 (18)	9 (53)	5 (29)	39.6 (20.9)	16.3 (20.7)	−23.4 (20.4)
Rotator cuff rupture *n* = 68 (39%)	13 (19)	41 (60)	14 (21)	45.6 (20.9)	17.8 (15.1)	−27.8 (22.5)
Glenohumeral instability *n* = 39 (22%)	3 (8)	30 (77)	6 (15)	21.9 (14.9)	12.5 (12.9)	−9.4 (16.9)
AC osteoarthritis *n* = 20 (11%)	2 (10)	16 (80)	2 (10)	43.6 (1.91)	16.5 (18.2)	−27.1 (21.4)
Glenohumeral osteoarthritis *n* = 29 (16%)	10 (34)	13 (45)	6 (21)	51.1 (22.0)	27.7 (21,].7)	−22.4 (19.4)
Frozen shoulder *n* = 3 (2%)	1 (33)	1 (33)	1 (33)	61.3 (16.6)	43.7 (44.1)	−17.7 (29.2)

^1^Patients with psychological disorders before and after shoulder surgery

^2^Patients with no psychological disorders before and after shoulder surgery

^3^Patients with psychological disorders before surgery and no psychological disorders after surgery and patients with no psychological disorders before surgery and new psychological disorders after surgery

### Minimal clinically important difference

The mean change scores per subgroup based on the functional anchor are presented in Table 3. The numbers of patients in the ‘unchanged’ and ‘worse’ categories were too small to calculate the mean change scores. The mean change score of the slightly improved group was used to determine the MCID of the DASH. The MCID was 13.0 [SD 20.7] in the group with symptoms of psychological disorders and 12.7 [SD 17.6] in the group with no symptoms of psychological disorders. We observed no difference (*p* = 0.559) in the MCID between the group with and the group without symptoms of psychological disorders.

**Table 3:** Anchor question function in group 1 and group 2

Functional anchor	No psychological disorders before and after surgery		Psychological disorders before and after surgery		*p* value
	*n* = 110		*n* = 32		
	Number (%) score	Change of DASH (mean; SD)	Number (%) score	Change of DASH (mean; SD)	
Completely recovered	45 (41%)	−28.3 (24.5)	4 (13%)	−45.5 (26.3)	*p* = 0.284
Much improved	44 (40%)	−24.3 (19.3)	16 (50%)	−20.9 (14.9)	*p* = 0.483
Slightly improved	14 (13%)	−12.7 (17.6)	6 (19%)	− 13.0 (21.7)	*p* = 0.978
Unchanged	5 (5%)	*	2 (6%)	*	*
Slightly worse	1 (1%)	*	1 (3%)	*	*
Much worse	1 (1%)	*	3 (9%)	*	*
Worse than ever	0 (0%)	*	0 (0%)	*	*

*Numbers too small to calculate

## Discussion

No difference in the MCID of the group with symptoms of psychological disorders was found compared to the group without symptoms of psychological disorders. Although symptoms of psychological disorders had a significant negative effect on the magnitude of the DASH score, it had no effect on the MCID in our study population. The MCID of the DASH score could therefore be used in all patients after shoulder surgery, irrespective of the presence of psychological symptoms.

Monitoring the effects of treatment is of well-recognized importance and is the foundation of modern evidence-based health care [10]. In our study population and in other studies [3–5], DASH scores were significantly worse in patients with psychological symptoms. This effect could not be explained by differences in age, gender, duration of symptoms or diagnosis but seems to reflect the negative influence of psychological symptoms on the DASH score. In order to interpret clinical outcome, the influence of psychological symptoms on the outcome measurement instrument should be taken into account [6].

Three clinical studies have investigated if preoperative psychological disorders were associated with functional outcomes after shoulder surgery [6, 27, 28]. They all showed that functional outcomes after shoulder surgery were not negatively influenced by preoperative psychological symptoms. Shoulder surgery resulted in a significant change in DASH scores after surgery in most patients, irrespective of the presence of psychological symptoms before surgery [6, 27, 28]. It is however important to know if a certain change in DASH score indicates the same perceived improvement in patients with and patients without psychological symptoms. We observed that the DASH change score which patients perceived to be important was not different in patients with and without psychological symptoms. This means that the MCID of the DASH score could be used in all patients after shoulder surgery, irrespective of the presence of psychological symptoms. Although there is debate in literature about the best measurement technique to calculate the MCID of the DASH score [26], the anchor-based mean change method is most frequently used [8–10, 12, 13]. There is no international consensus on the optimal cut-off point on an anchor; however, we think that the slightly improved group best reflects a minimally important change opposed to the much improved group. Previously published MCID of the DASH score ranges from 10 to 13 [10–13]. All these studies were performed in heterogeneous study populations, with study samples ranging from 53 to 361 patients.

There are some limitations that have to be mentioned. First, the study sample was relatively small, especially in the group of patients with psychological symptoms before and after surgery. The perceived improvement of pain and function 12 months after shoulder surgery was good to excellent in most patients, leaving a small number of patients with unchanged or worse clinical results. Further studies with larger study samples should include more patients reporting inferior clinical outcomes, for example, including patients treated conservatively or patients after shoulder surgery but with a shorter follow-up period.

Second, the 4DSQ questionnaire is a tool to identify psychological symptoms. However, having significant psychological symptoms is not the same as having a psychological illness. Psychological disorders have to be diagnosed by a psychologist or psychiatrist using DSM-V criteria. We did not account for the use of antidepressive medication and if patients are treated by a psychologist or psychiatrist during the study period.

Third, we used a heterogeneous population for calculation of the MCID. We included a heterogeneous patient population with shoulder complaints, with different diagnoses, operations, levels of pain and functional disability and duration of symptoms. Psychological symptoms were observed in all various shoulder diagnoses before surgery. There is no evidence in the literature that the MCID differs among (sub)populations with different diagnoses and surgical or non-surgical treatment, but it has been suggested that this should be evaluated [26, 29]. We could not perform subgroup calculations in our study population because the subgroups would be too small. The advantage of using a heterogeneous cohort is that it provides a MCID estimation that can be used in all kinds of shoulder disorders. Future studies should investigate if and how the MCID varies among shoulder subgroups. We only studied the MCID and did not measure the smallest detectable change (SDC). In order to calculate the SDC, two measurements of a study population at two time periods close together are needed; we only measured patients before and 12 months after surgery. As the MCID observed in the two groups of our study population (MCID 13) are within the range of previously published MCID of the DASH score (range from 10 to 13) [10–14], we assume that the SDC of our study population might be also in the range of these reports (range from 10 to 16) [10, 12–14].

## Conclusion

Symptoms of psychological disorders in patients treated with shoulder surgery seem to have a negative effect on the magnitude of the DASH score but did not influence the MCID of the DASH score. This means that the MCID of the DASH score could be used in all patients after shoulder surgery, irrespective of the presence of psychological symptoms. The DASH score could be used in future studies to assess the influence of psychological factors on the clinical outcome of treatment.

## Abbreviations

4DSQ: Four-Dimensional Symptom Questionnaire
DASH: Disabilities of the Arm, Shoulder and Hand questionnaire
MCID: Minimal clinically important difference
